# Retraction: Epoxy coating with embedded self-healing networks formed by nanogel particles

**DOI:** 10.1039/d5ra90074c

**Published:** 2025-06-11

**Authors:** Ayman M. Atta, Hamad A. Al-Lohedan, Khalid A. Al-Haddad

**Affiliations:** a Chemistry Department, College of Science, King Saud University Riyadh 11451 Saudi Arabia aatta@ksu.edu.sa hlohedan@ksu.edu.sa Mr.alhddad@gmail.com +966 114675998 +966 561557975; b Petroleum Application Department, Egyptian Petroleum Research Institute Nasr City 11727 Cairo Egypt +20 222747433 +20 222747917

## Abstract

Retraction of ‘Epoxy coating with embedded self-healing networks formed by nanogel particles’ by Ayman M. Atta *et al.*, *RSC Adv.*, 2016, **6**, 41229–41238, https://doi.org/10.1039/C6RA03523J.

The Royal Society of Chemistry hereby wholly retracts this *RSC Advances* article due to concerns with the reliability of the data.

The TEM data in Fig. 1a, b, d and f each have multiple repeating sections that appear very similar. The authors were not able to provide the original raw data nor satisfactorily explain the concerns.

Given the significance of these concerns, the findings presented in this paper are no longer reliable.

Ayman M. Atta and Hamad A. Al-Lohedan were informed of the decision to retract this article. We were unable to contact Khalid A. Al-Haddad. Ayman M. Atta has not agreed with the decision and Hamad A. Al-Lohedan has not responded.

Signed: Laura Fisher, Executive Editor, *RSC Advances*

Date: 28th May 2025

